# Rulers of the Open Sky at Risk: Climate-Driven Habitat Shifts of Three Conservation-Priority Raptors in the Eastern Himalayas

**DOI:** 10.3390/biology14101376

**Published:** 2025-10-08

**Authors:** Pranjal Mahananda, Imon Abedin, Anubhav Bhuyan, Malabika Kakati Saikia, Prasanta Kumar Saikia, Hilloljyoti Singha, Shantanu Kundu

**Affiliations:** 1 Animal Ecology and Wildlife Biology Lab, Department of Zoology, Gauhati University, Jalukbari 781014, India; 2 Wildlife Ecology Lab, Department of Zoology, Bodoland University, Kokrajhar 783370, India; 3 Department of Environmental Science, Tezpur University, Napaam 784028, India; 4 Centre for Wildlife Research and Biodiversity Conservation, Bodoland University, Kokrajhar 783370, India; 5 Ocean and Fisheries Development International Cooperation Institute, College of Fisheries Science, Pukyong National University, Busan 48513, Republic of Korea

**Keywords:** birds of prey, northeast India, habitat loss, species distribution modeling, conservation

## Abstract

**Simple Summary:**

Raptors, as apex predators, serve as valuable bioindicators for assessing the impacts of climate change because of their specialized ecological traits, which render them particularly susceptible to environmental alterations. Globally, raptors are experiencing significant conservation concerns, with approximately 52% of species exhibiting declining populations and 18% being classified as threatened. Despite this, the effect of climate change on raptors is poorly studied in the Eastern Himalayan region. Three species, *Falco severus*, *Gyps tenuirostris* and *Haliaeetus leucoryphus,* were selected based on their conservation status in the region. This study provides a comprehensive assessment of climate change impacts on raptors in the northeastern part of the Eastern Himalayas, utilizing ensemble species distribution modeling for the projected periods 2041–2060 and 2061–2080. The future projections indicate a substantial decline in suitable habitats: *Falco severus* is projected to lose 33–41%, *Gyps tenuirostris* may lose 53–96%, and *Haliaeetus leucoryphus* is anticipated to experience a loss of approximately 94–99% of its suitable habitats.

**Abstract:**

Raptors, being at top of the food chain, serve as important models to study the impact of changing climate, as they are more vulnerable due to their unique ecology. They are vulnerable to extinction, with 52% species declining population and 18% are threatened globally. The effect of climate change on raptors is poorly studied in the Eastern Himalayan region. The present study offers a complete investigation of climate change effects on the raptors in the northeast region of the Eastern Himalayas, employing ensemble species distribution modeling. The future predictions were employed to model the climate change across two socioeconomic pathways (SSP) i.e. SSP245 and SSP585 for the periods 2041–2060 and 2061–2080. Specifically, five algorithms were employed for the ensemble model, viz. boosted regression tree (BRT), generalized linear model (GLM), multivariate adaptive regression splines (MARS), maximum entropy (MaxEnt) and random forest (RF). The study highlights worrying results, as only 10.5% area of the NE region is presently suitable for *Falco severus*, 11.4% for the critically endangered *Gyps tenuirostris*, and a mere 6.9% area is presently suitable for the endangered *Haliaeetus leucoryphus*. The most influential covariates were precipitation of the driest quarter, precipitation of the wettest month, and temperature seasonality. Future projection revealed reduction of 33–41% in suitable habitats for *F. severus*, *G. tenuirostris* is expected to lose 53–96% of its suitable habitats, and *H. leucoryphus* has lost nearly 94–99% of its suitable habitats. Such decline indicates apparent habitat fragmentation, with shrinking habitat patches.

## 1. Introduction

In recent years, global ecological stability has faced considerable disorder due to the accelerating impacts of climate change and anthropogenic-induced stress [1]. This accelerated pace of climate change and its adverse impacts on species and their populations have emerged as a global conservation concern [2]. It alters the ecosystem structure and functioning of the habitats used by the faunal species [3]. Furthermore, human-induced land use changes have also led to a significant decline in overall species diversity and habitat quality, particularly within avian communities [4]. Therefore, understanding species habitats and the impacts of climate change has become imperative for the conservation of vulnerable and threatened taxa worldwide.

Raptors are important models for examining the effects of climatic change and habitat loss, as they occur worldwide, perform vital ecological roles and serve as surrogate species for preservation of biodiversity [5,6,7]. They face a higher risk of extinction compared to other avian groups due to their unique ecology and life history traits [8]. They are long-lived apex predators with large home ranges and feed on a diverse array of prey items from insects to mammals, and so are influenced by climate change effects on the lower trophic levels [9,10]. Hence, the raptors may amplify climate change effects by affecting the ecosystem disproportionately [11]. Also, raptors are vulnerable to extinction, with 52% species existing in declining population trend and 18% being threatened globally; additionally, the populations of 38% of Least Concern species are declining [8]. Apart from various threats, raptors are also highly threatened by habitat alteration [12,13] and changing climate [14,15].

The geographic range of a raptor is primarily governed by the intersection of thermal niche, preferred preys and suitable nesting ground [2]. The range shift trend of raptors is predominantly towards higher elevation, but the magnitude and direction of the range shift can vary between species owing to their life story, food habits and their breeding and wintering grounds [2,16,17]. These shifts might have a negative effect on the ecological structure, resulting in conservation consequences [2]. As raptors shift their ranges, new assemblages of species may emerge, leading to competition for resources, and changing prey–predator dynamics [18]. The changes in climatic conditions have also resulted in range shifts in diseases and pathogens, thus disrupting the local disease ecology [19,20]. The change in disease ecology due to climate change will further result in increased mortality of raptors and rapid population decline [2]. Climate change is shifting the distribution of diseases, parasites, and ectoparasites northward, disrupting host–pathogen dynamics and exposing young raptor populations to novel infections with potentially severe impacts on their survival [21].

Moreover, climatic shifts have affected the annual breeding cycles and phenology of raptors, with them now exhibiting early phenology [22,23]. There is a shift in breeding phenology due to climate change, particularly at higher altitudes, though responses may differ by species, diets and locations [24]. These responses may vary from larger clutches and improved survival to reduced success. Climate change profoundly influences raptor migratory patterns [25,26]. Raptors are altering migration schedules, delaying autumn departures while advancing spring arrivals [27,28]. Additionally, many species show migratory short-stopping wintering nearer their breeding ranges under changing environmental conditions. Change in climate also affects the thermal dynamics of soaring raptors [29,30], with resultant extreme weather events proving detrimental to endangered and restricted-range species, often causing significant population declines [31,32]. About 25% population of raptors significantly declined after severe hurricane [33,34].

According to research and conservation priority index [35], *Falco severus* (Horsfield, 1821), *Gyps tenuirostris* (Gray, 1844) and *Haliaeetus leucoryphus* (Pallas, 1771) are the three priority raptors for conservation that breed in northeastern India. However, currently, there are no comprehensive breeding and nesting data for these species in the region. The species *Falco severus* (Oriental hobby) has an IUCN range extending from India to Bhutan and Nepal, through Myanmar, China, Thailand, Laos, Vietnam, Cambodia, Philippines, Indonesia, and continuing eastwards to Papua New Guinea and Solomon Islands [36]. It was earlier considered to breed but recorded now as wintering in the Western Ghats, and there is no occurrence record in the 20th and 21st centuries [37]. The IUCN range of *Gyps tenuirostris* (Slender-billed vulture) extends from India through north of the Gangetic plain, west to Himachal Pradesh and Haryana, to southern West Bengal, eastwards through the Assam plains, to southern Nepal and Bangladesh [38]. As per IUCN, *Haliaeetus leucoryphus* (Pallas’s fish-eagle) ranges from India (with breeding grounds in Assam and Uttarakhand). Outside India, it is known to breed in Bangladesh, Nepal and Bhutan. The non-breeding range extends from Northern Himalayas towards Kazakhstan, Russia and Mongolia [39]. All three raptor species have overlapping breeding ranges in the northeast, and belong to different trophic niches [40,41]. The *F. severus* typically nests in trees and occurs in forests with clearings, foothill forests, and occasionally plantations [41]. In contrast, *G. tenuirostris* is primarily associated with open-country habitats, foothills, and areas near human settlements, where it builds nests on tall trees. The major threats to this species include exposure to the NSAID diclofenac, deliberate poisoning, habitat loss, and food scarcity [41]. The *H. leucoryphus* generally inhabits riverine systems and flooded wetlands, utilizing large nearby trees for nesting. The major threats to this species identified are habitat degradation, water pollution, and overfishing [41].

The Himalayas are the best place to study climate change impacts, as the region has varied climatic zones and habitat, with diverse flora and fauna adversely affected by climate change [42]. The northeast region of the Eastern Himalayas is home to about 54 species of diurnal raptors, which accounts for about 77% of the total raptors found in the Indian subcontinent [43]. Out of these, as per IUCN, three species are Critically Endangered, two are Endangered, and 4 species are Near Threatened. There has been no status assessment of raptors in the region. According to Mahananda et al. [35], this region has only 4% of the studies conducted in India. Many raptor species in this region with Least Concern status (IUCN) have declining population trends [44]. This region also falls within the Circum Himalayan Corridor of the Central Asian Flyways, which is used by many migrating raptors [45,46]. The terai grasslands and forests of the Eastern Himalayas represents areas of highest species vulnerability, and the region is facing extensive habitat alterations [47]. Although ornithological studies have flourished in the region in recent times, raptors remain poorly studied [35]. Also, the region has suffered significant loss of bird habitats, with 60% of all tree cover loss between 2001 and 2023 (www.globalforestwatch.org, accessed on 5 June 2025) and conversion to monoculture plantations. Hence, to study the impact of climate and species range shifts, species distribution models (SDMs) are commonly used globally [48]. SDMs recognize the environmental envelope of species presence and forecast changes in their ranges on the basis of future climatic conditions, serving as common conservation prioritizing tool [49,50]. Recent studies have highlighted alarming trends in raptor population decline due to habitat loss [2,51,52]. Furthermore, changes in climatic conditions have forced the raptors to shift to suitable habitats or face extinction [2,53]. Taking this into account, the present study aims to (i) assess the impact of climate change on the three selected raptor species (*F. severus*, *G. tenuirostris* and *H. leucoryphus*) with the use of species distribution model (sdm) under current and future climate scenarios, (ii) and to evaluate the extent of habitat loss of the raptors in NE India.

## 2. Materials and Methods

### 2.1. Study Area

**Figure 1:**
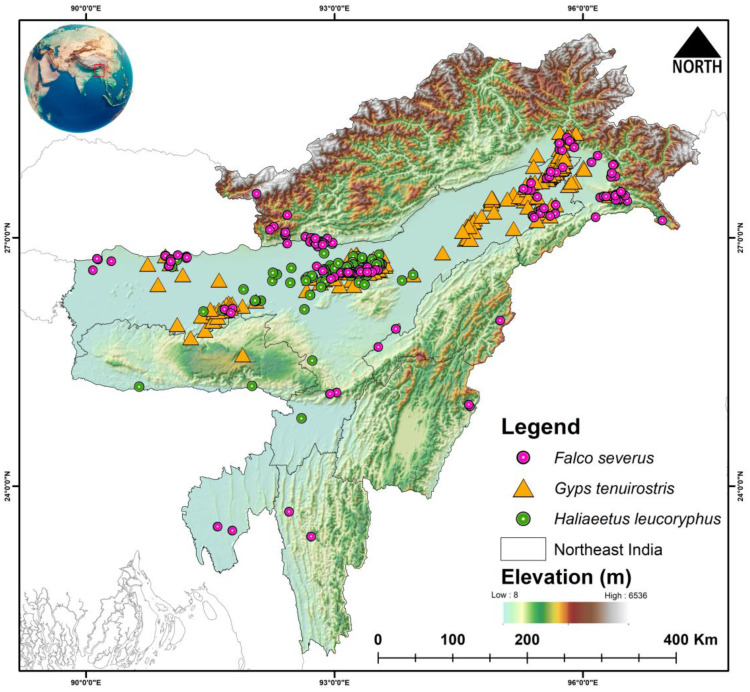
Location occurrences of three raptor species, i.e., Falco *severus*, *Gyps tenuirostris*, and *Haliaeetus leucoryphus* within the study area (Northeast India), compiled from primary field surveys and secondary sources. The map was created in ArcGIS v10.6, and the inset globe was manually edited in Adobe Photoshop CC.

The Northeast region of India forms a major portion of the Himalaya and Indo-Burma biodiversity hotspots [54] (Figure 1). The region has three broadly distinguished areas: the Eastern Himalayas in the north [55,56], and NE hills to the south, separated by the Brahmaputra River basin in between [55,57]. The region exhibits a unique biogeography due to multiple reasons such as its location at the junction of biogeographic realms [55], paleoclimatic history, geology, etc. It is characterized by diverse habitats from tropical and montane forest to snow-capped mountains, including elevation range from 100 msl to 7000 msl.

The selected study area, i.e., northeast region of the Eastern Himalayas, encompasses the IUCN distribution extent of the three raptors breeding in the region. This method of delineating the suitable extent of the species enables us to predict the suitable habitats accommodating all three species. The field surveys (permission No. WL/FG.31/Pt/Technical Committee/2018dt. 24 January 2019) were conducted in the northeastern India in Assam during the period 2018–2022; the occurrence coordinates were obtained with handheld Garmin GPS (Garmin, Gurgaon, India) and photographs were captured with Nikon Coolpix P510 (Nikon, Gurgaon, India) and Nikon P600 camera (Nikon, Gurgaon, Haryana). Furthermore, secondary occurrence records were also incorporated to expand the overall distribution range for all species. These records were obtained from the IUCN Geospatial Conservation Assessment Tool (GeoCAT), which compiles data from multiple reputable repositories and is widely recognized as a reliable source [58]. The dataset contains diverse record types, including direct field observations, captive individuals, and historical museum specimens. Since not all of these accurately reflect the present habitat or environmental envelope of the species, records associated with museum specimens and captive individuals were excluded. Therefore, only direct human observations were retained to enhance the reliability of model predictions. To develop the ensemble models, occurrences from both field surveys and secondary sources were utilized. From field surveys, five occurrence points were used for *Falco severus* and six for *H. leucoryphus*. From secondary data sources, a total of 145 occurrence points for *Falco severus* [59], 104 points for *G. tenuirostris* [60], and 108 points for *H. leucoryphus* [61] were used to develop the ensemble models, selecting spatially independent occurrence points using the SDM Toolbox v2.4 [62]. A resolution of 1 km^2^ was used to conduct the spatial correlation between occurrences, equal to pixel size in the raster data, thus avoiding over-fitting of the model [63].

### 2.2. Covariates for Suitable Habitat Evaluation

The 19 bioclimatic variables were obtained from the WorldClim database to represent the diverse climatic envelopes associated with the species (Table S1) [64]. Further, the topographic data such as aspect, slope and elevation, were attained from the Diva-Gis database at 90 m resolution [65]. All selected variables were resampled to a spatial resolution of 30 arcseconds (~1 km^2^) using ArcGIS 10.6. The spatial multicollinearity among the variables was assessed in VisTrails software using the SAHM package v2.2.3 [66]. The variables with pairwise correlation coefficients below 0.8 based on Pearson, Spearman, and Kendall correlation coefficients were retained for further analysis (Figures S1–S3). Following this correlation analysis, a final set of 10 variables was selected for the model

The future predictions were employed to model the climate change across two socioeconomic pathways (SSP)—SSP245 and SSP585, for the periods 2041–2060 and 2061–2080. The SSP245 represents an intermediate scenario in which global development follows a “middle-of-the-road” trajectory, with moderate population growth, technological progress, and policies that gradually stabilize greenhouse gas (GHG) emissions by the end of the century, despite significant challenges [67]. Alternatively, SSP585 depicts a fossil fuel-intensive world with rapid population growth, high energy demand, limited climate policies, and continuously rising GHG emissions, leading to stronger climate forcing and associated impacts on ecosystems [68]. Additionally, the Hadley Centre Global Environment Model in Global Coupled Configuration 3.1 (HadGEM3-GC31 LL) of the sixth Coupled Model Intercomparison Project (CMIP6) was utilized during the study [69]. This general circulation model (GCM) was selected based on its known effective performance in South and Southeast Asia, and its capability to indicate time-based variations and correctly representing temperature dispersal, as observed in earlier studies [63,70,71,72,73].

### 2.3. Species Distribution Modeling

An ensemble modeling approach was employed for the three species, integrating multiple algorithms to combine their individual strengths. This method effectively captures the diverse ecological and statistical relationships that shape species distributions. Specifically, five algorithms were employed for the ensemble model, viz., boosted regression tree (BRT), generalized linear model (GLM), multivariate adaptive regression splines (MARS), maximum entropy (MaxEnt) and random forest (RF) [73,74]. Furthermore, the pseudo-absence points were randomly generated within the study area to enable fitting of the ensemble SDM. The Software for Assisted Habitat Modeling (SAHM) software v2.2.3 was used to run the ensemble model using the VisTrails workflow system [74,75]. The models generated continuous habitat suitability maps, with predicted values ranging from 0 (unsuitable) to 1 (highly suitable). Furthermore, for generating the binary presence–absence maps, these continuous outputs were subsequently thresholded using the sensitivity-equals-specificity (SES) criterion. This criterion balances omission and commission errors, offering an objective and widely applied cutoff in species distribution modeling. It helps minimize systematic overprediction or underprediction of suitable habitats and enables consistent comparisons across multiple algorithms. The exclusion criteria for the selected models were based on the area under curve (AUC) threshold value of 0.75 [76,77]. The ensemble map was produced scaling from 0 to 5, where the model agreement was denoted by each pixel, with the value of 5 signifying unanimity in agreement across all the models, facilitating the investigation of habitat conformation. Furthermore, to evaluate models’ performance, different metrics such as area under curve (AUC), true skill statistic (TSS), Cohen’s Kappa, proportion correctly classified (PCC), and sensitivity and specificity were measured for both training data and cross-validation (*n* = 10) [78,79].

### 2.4. Habitat Quality Assessment

In order to assess the quantitative and qualitative features of the suitable habitat areas in present and future climate set-ups, different class-level metrics were applied with FRAGSTATS software version 4.2.1 [80,81]. It analyzes the spatial patterns in landscapes and provides useful metrics to evaluate landscape characteristics. For this study, the metrics used were as follows: total number of patches (NP), largest patch index (LPI), total edge (TE), landscape shape index (LSI) and aggregate index (AI). NP, LPI and TE provide detailed analysis of the geometry, such as patch size and edge features. LSI provides information on the complexity and irregularity of the shape of patches, while AI evaluates the distance of the patches from one another within the landscapes. These metrics are significant in highlighting the effects of transformation in suitable ranges within a landscape [82,83]. These metrics were used to evaluate the habitat structures and assess the extent of fragmentation in the selected area in present and future climate scenarios [84,85].

## 3. Results

### 3.1. Model Performance

All models performed strongly on both training and cross validation data (Figures S4–S6, Table 1). The models showed an AUC value above 0.80 for training and cross-validation datasets for all three species (Figures S4–S6, Table 1). The maximum training AUC value was attained by the GLM algorithm for *F. severus* (AUC = 0.951), while the BRT model generated the highest training AUC both for *G. tenuirostris* (0.985) and *H. leucoryphus* (0.988). In terms of model performance during cross-validation, the maximum AUC value was generated by RF for *F. severus* (AUC = 0.891), while for *G. tenuirostris* the model RF attained highest value (AUC = 0.937) and for *H. leucoryphus*, both BRT and RF models generated highest AUC value during cross-validation (AUC = 0.958). The highest ΔAUC value was seen in the GLM for *F. severus* (0.122) indicating model overfitting, while for *G. tenuirostris* the highest ΔAUC was achieved by the BRT model (0.068), and for *H. leucoryphus* the BRT and RF models produced the highest ΔAUC value (0.038). On the other hand, the RF model showed the lowest ΔAUC values across all the three species. The good performance of the models was also supported by the valuation metrics such as TSS, PCC, Kappa, Sensitivity and Specificity, which yielded maximum values, thus supporting the predictive consistency of the ensemble modeling approach.

### 3.2. Variable Importance

The model for *F. severus* showed that across the five models, on an average (μ), the variable precipitation of coldest quarter (bio19) was the highest contributor (29.02%) (Table 2). Elevation (23.66%) was the variable most contributing topographic to the models (Table 2). Similarly, the ensemble model for *G. tenuirostris* indicated elevation as the highest contributor (37.14%), while temperature seasonality (bio4) was the highest bioclimatic contributor (23.18%). For *H. leucoryphus*, the most influencing habitat variable was elevation (54.02%), and precipitation of the driest month (bio14) was the highest contributing bioclimatic variable (16.41%) (Table 2).

### 3.3. Habitat Suitability

**Figure 2:**
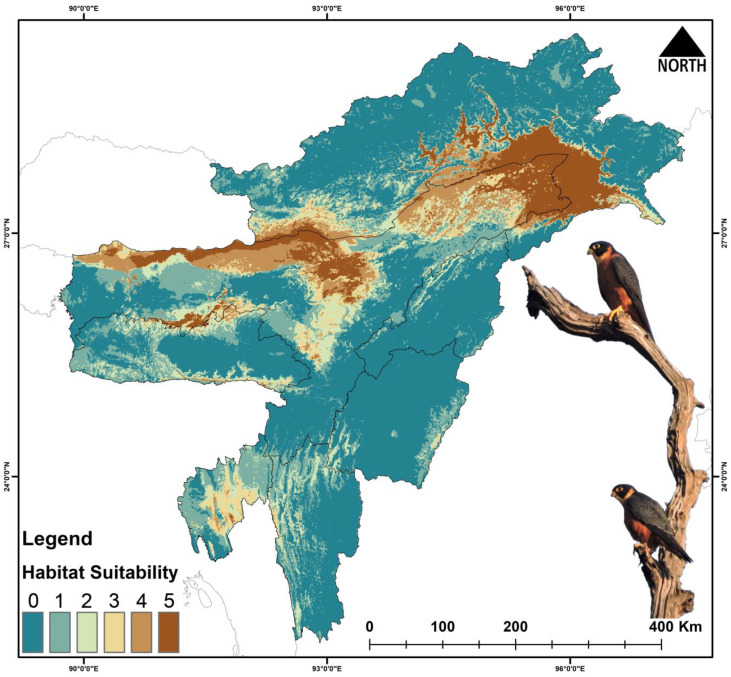
Habitat suitability of *Falco severus* in Northeast India under the present scenario. The scale (0–5) represents the level of ensemble model agreement, where ‘0’ indicates no model agreement for a pixel and ‘5’ indicates full agreement across all models, denoting habitat suitability. The inset photograph of the species was taken by the first author (P.M.).

The total area selected for the study is about 255,083 km^2^. Out of this area, for *F. severus*, the present suitable area is 26,801 km^2^, which is 10.5% of the total area (Figure 2, Table 3 and Table S2). During the years 2041–2060 (SSP245), the suitable area is reduced to 17,109 km^2^, and during 2061–2080 (SSP245) the area is 9231 km^2^. Similarly, the suitable area during the period 2041–2060 (SSP585) is 12,184 km^2^, and during 2061–2080 (SSP585) the area is further reduced to 8389 km^2^. The percentage of suitable area loss from the present area is 36.16% during 2041–2060 (SSP245), and the loss during 2061–2080 (SSP245) is 65.55%. Similarly, the percent suitable area loss during 2041–2060 (SSP585) is 54.53% and there is 68.69% loss during 2061–2080 (SSP585).

**Figure 3:**
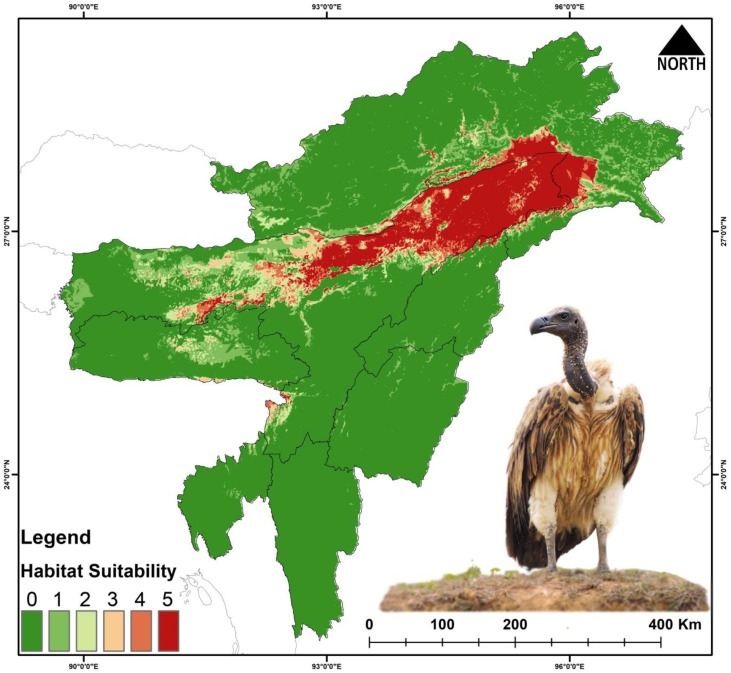
Habitat suitability of *Gyps tenuirostris* in Northeast India under the present scenario. The scale (0–5) represents the level of ensemble model agreement, where ‘0’ indicates no model agreement for a pixel and ‘5’ indicates full agreement across all models, denoting habitat suitability. The inset photograph of the species was taken by the Nilutpal Mahanta.

For *G. tenuirostris*, 29,297 km^2^ (11.4%) is currently suitable (Figure 3, Table 3 and Table S2). During the years 2041–2060 (SSP245), the suitable area is reduced to 13,731 km^2^, and during 2061–2080 (SSP245) the area is 989 km^2^. Similarly, the suitable area during the period 2041–2060 (SSP585) is 6160 km^2^, and during 2061–2080 (SSP585) the area is further reduced to 919 km^2^. The percentage of suitable area loss from the present area is 53.13% during 2041–2060 (SSP245), and the loss during 2061–2080 (SSP245) is 96.62%. Similarly, the percent suitable area loss during 2041–2060 (SSP585) is 78.97%, and there is 96.86% loss during 2061–2080 (SSP585).

**Figure 4:**
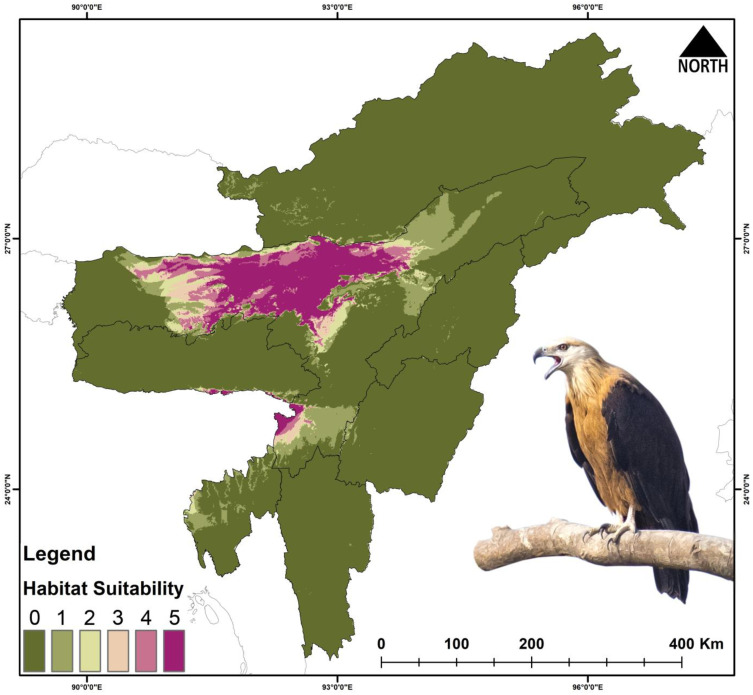
Habitat suitability of *Haliaeetus leucoryphus* in Northeast India under the present scenario. The scale (0–5) represents the level of ensemble model agreement, where ‘0’ indicates no model agreement for a pixel and ‘5’ indicates full agreement across all models, denoting habitat suitability. The inset photograph of the species was taken by Nilutpal Mahanta.

The present suitable area for *Haliaeetus leucoryphus* is 17,808 km^2^, which is 6.9% of the total area (Figure 4, Table 3 and Table S2). During the years 2041–2060 (SSP245), the suitable area is reduced to 1021 km^2^, and during 2061–2080 (SSP245) the area is 444 km^2^. Similarly, the suitable area during the period 2041–2060 (SSP585) is 419 km^2^, and during 2061–2080 (SSP585) the area is further reduced to 119 km^2^. The percentage of suitable area loss from present area is 94.26% during 2041–2060 (SSP245), and the loss during 2061–2080 (SSP245) is 97.5%. Similarly, the percent suitable area loss during 2041–2060 (SSP585) is 97.64%, and there is 99.33% loss during 2061–2080 (SSP585).

**Figure 5:**
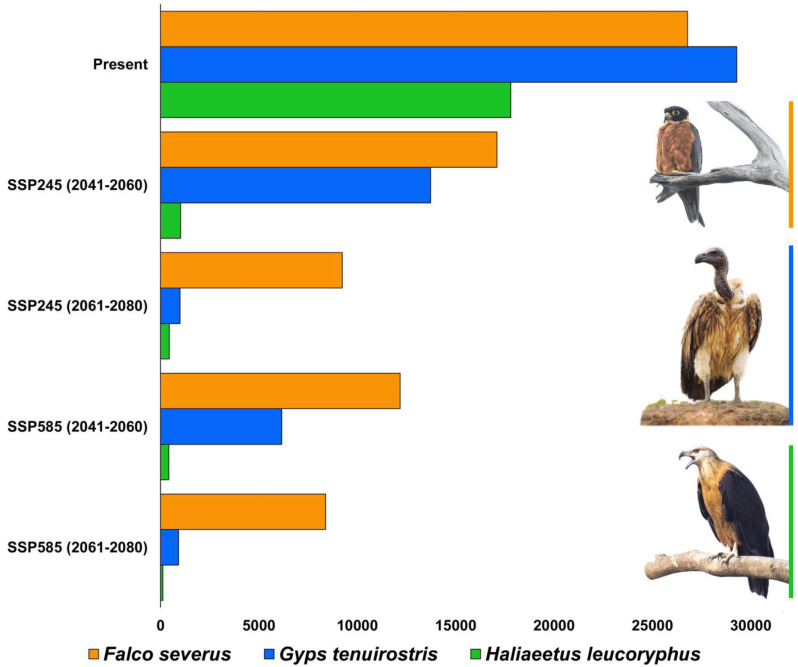
Bar chart showing the habitat suitability (in km^2^) of three raptor species, *Falco severus*, *Gyps tenuirostris*, and *Haliaeetus leucoryphus*, within the study area (Northeast India) under present and future climatic scenarios. Inset photographs are credited to Wich’yanan Limparungpatthanakij (*Falco severus*) and Nilutpal Mahanta (*Gyps tenuirostris* and *Haliaeetus leucoryphus*).

**Figure 6:**
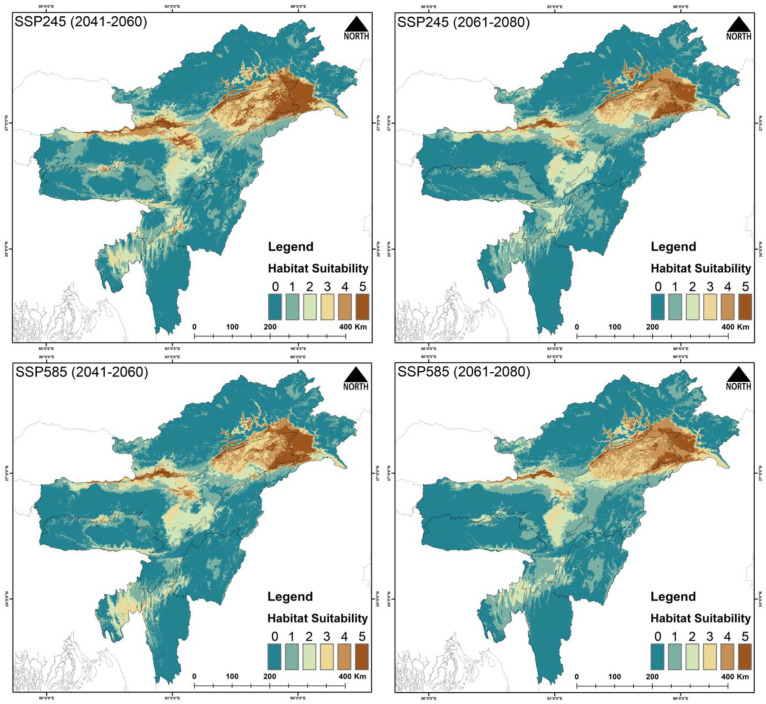
Habitat suitability of *Falco severus* in Northeast India under future climatic scenarios SSP245 and SSP585 for two time periods: 2041―2060 and 2061―2080. The scale (0―5) indicates ensemble model agreement, where ‘0’ denotes no model agreement for a pixel and ‘5’ denotes full agreement across all models, indicating high habitat suitability.

**Figure 7:**
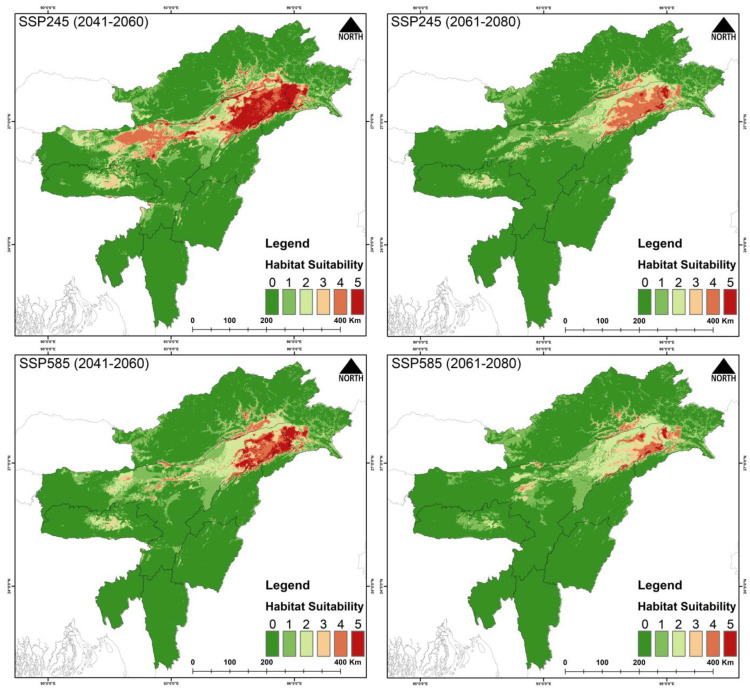
Habitat suitability of *Gyps tenuirostris* in Northeast India under future climatic scenarios SSP245 and SSP585 for two time periods: 2041―2060 and 2061―2080. The scale (0―5) indicates ensemble model agreement, where ‘0’ denotes no model agreement for a pixel and ‘5’ denotes full agreement across all models, indicating high habitat suitability.

**Figure 8:**
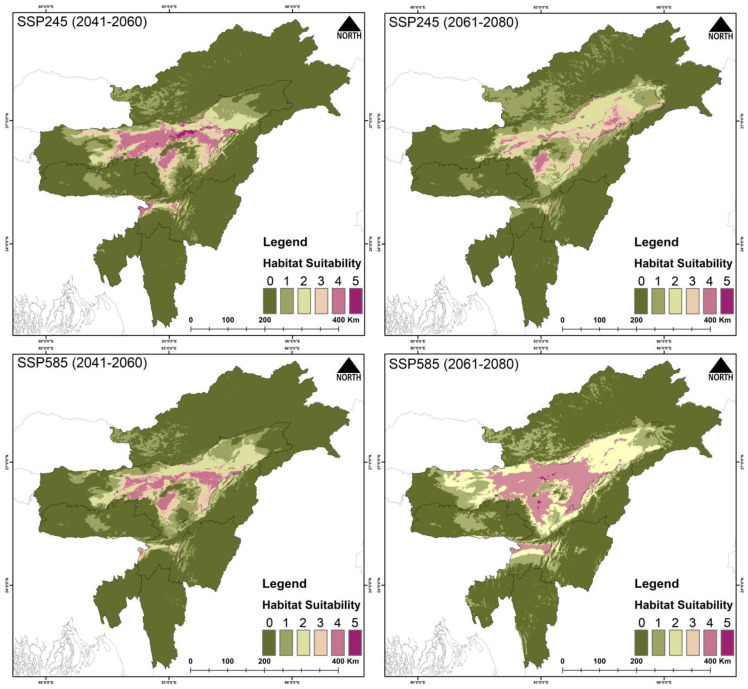
Habitat suitability of *Haliaeetus leucoryphus* in Northeast India under future climatic scenarios SSP245 and SSP585 for two time periods: 2041―2060 and 2061―2080. The scale (0―5) indicates ensemble model agreement, where ‘0’ denotes no model agreement for a pixel and ‘5’ denotes full agreement across all models, indicating high habitat suitability.

The future climate projection indicated vast loss of habitats with 36–68% loss in the habitats for *Falco severus* under SSP245 and SSP585 scenarios (Figure 5 and Figure 6, Table 3 and Table S2). For *G. tenuirostris*, the habitat loss in future climate scenarios was 53–96% (Figure 5 and Figure 7, Table 3 and Table S2). The most extreme loss of habitat under future climate was observed for *Haliaeetus leucoryphus*, which suffered 94–99% loss under SSP245 and SSP585 scenarios (Figure 5 and Figure 8, Table 3). All three species showed severe reductions in suitable habitat area in present and future climate (Figure 5, Table S2).

### 3.4. Quality of the Suitable Habitat

The severe loss of habitat of the raptors has resulted in significant changes to the geometry of the area. Fragmentation analysis suggests significant changes in the geometry of the area due to the severe loss of suitable habitats. For *F. severus*, there was a 33% decrease in the number of patches (NP) in SSP245, and 41% in SSP585 scenarios (Table 4). There was a significant reduction in the suitable areas for *F. severus*, with about 64.64% decrease in patch sizes (LPI) in SSP245 and 79.37% in SSP585. Also, there was a 70.20% decrease in number of fragmented patches in SSP245 and 77.25% in SSP585 scenarios within the suitable habitats of *G. tenuirostris*, with a 98.45% reduction in patch sizes (LPI) in SSP245 and 99.04% decrease in SSP585 scenarios. For *H. leucoryphus*, there was a 77.05% decrease in number of patches (NP) in SSP245 and 90.16% in SSP585 climate scenarios. There was also a severe reduction in patch size (LPI) by 99.96% in SSP245 and by 99.98% decrease in SSP585 scenarios (Table 4). The metrics indicating the patch proximity (AI) showed a noteworthy decline, while edge structures (TE and LSI) also showed an increase in edge shape complexity for all the three species (Table 4).

## 4. Discussion

### 4.1. Present Suitable Habitats

The model outputs have highlighted the major influence of climate change on the raptors’ occurrence and distribution in Northeast India. Among the three species, *G. tenuirostris* has the broadest suitable habitat under current climatic conditions, spreading over 29,297 km^2^, followed by *F. severus*, which has an area of 26,801 km^2^, while *H. leucoryphus* has the lowest suitable habitat area of 17,808 km^2^. Out of the total study area, the limited availability of suitable habitats for *F. severus* (10.5%), *G. tenuirostris* (11.4%), and *H. leucoryphus* (6.9%) highlights noteworthy spatial restraints within the study area. These results suggest likely habitat loss and fragmentation susceptibility, probably driven by anthropogenic causes. The highly suitable areas for *F. severus* were observed in Assam and Arunachal Pradesh. In Assam, Tinsukia, Dibrugarh, Golaghat, Biswanath, Sonitpur, Kamrup districts, and the northern BTR region showed high suitability areas, while Changlang, Lohit and Lower Dibang Valley districts of Arunachal showed many suitable areas. For *G. tenuirostris*, the high suitability area extends from Biswanath, Sonitpur and Golaghat districts to throughout the upper Assam area, including Changlang, Lower Dibang Valley, Namsai and Lohit districts of Arunachal Pradesh. Our result aligns with a study conducted on *G. leucoryphus* in Arunachal Pradesh [86]. The present suitable area of *H. leucoryphus* extends from middle Assam to the lower Assam, including the districts Golaghat, Biswanath, Sonitpur, Nagaon and BTR regions. The current predictive distribution evaluation also concerningly suggests a about 50–90% loss of suitable habitats in projected future climatic conditions. The expected reduction in suitable habitats has also been validated by other studies on raptors globally [87,88].

The predictors responsible for such effects are bioclimatic and topographic. The bioclimatic variables such as precipitation of the driest quarter, precipitation of the wettest month, and temperature seasonality were the most influencing factors. Climate predictive modeling studies have suggested that raptors in tropical regions have a multidirectional shift in their range [2,88]. These raptors track precipitation variations rather than temperature alone, as fluctuations in precipitation regimes highly influence the breeding success in the tropical regions [88]. Precipitation exerts differential effects on the abundance and survival of various species within the genus *Falco*, as observed by other researchers [89]. Increased precipitation has been directly associated with higher nestling mortality in falcons [90]. Studies have shown that temperature has been linked to brood loss in raptors [91], and temperature has also been linked to delayed migration [92] and to prey availability [2]. Heavy precipitation has been seen to increase juvenile mortality in raptors [92], decrease survival rate in nests [93], and reduce prey supply to the nest [94]. Thereby, precipitation is seen as a vital factor for the survival of raptors, as also observed in our study. Similarly, elevation was found to be the highly influential variable driving the distribution of *G. tenuirostris* (37.1%) and of *H. leucoryphus* (50.0%). Our findings draw similarity with Biju et al. (2024) [86], who observed elevation as the most influencing variable for the occurrence of *Gyps tenuirostris*. The study suggested that the species prefers a low-elevation foothill belt with high temperature and high precipitation.

The habitat suitability of raptors in the eastern Himalayas is mainly governed by elevation and temperature-related variables, showing the region’s complex topography and climate [95]. On the contrary, the main predictors in African savannas are precipitation and rainfall seasonality, which influence prey richness and open habitats crucial for raptors [96]. In temperate Europe, habitat suitability models are dominated by topographic variety, food availability, and precipitation, highlighting the importance of landscape patchiness [97]. Other South Asian regions exhibit habitat suitability for raptors that is influenced by a combination of elevation gradients, diverse habitat types such as forests and wetlands, and anthropogenic factors including proximity to human refuse sites. These environmental and human-related variables collectively shape raptor distribution patterns by affecting resource availability and habitat quality [98]. The suitability of raptor habitats is consistently influenced by climatic and topographic factors. However, the significance of variables such as rainfall, land use, and human-induced changes differs considerably across regions [99].

### 4.2. Future Climatic Predictions

In future climatic projections (under SSP245 and SSP585), it was observed that *F. severus* has lost 36–68% of its suitable habitats. Also, the quality of the habitat has degraded considerably, with a 33% decrease in the number of habitat patches in SSP245 and 41% in SSP585 scenarios (Table 4). Subsequently, there was a 64.64% reduction in the patch size, and increased distance from one patch to another. This raptor, being a species of forested foothills, utilizes tall trees to build nests in suitable forest habitats in the region [36,43]. The current and future trends in loss of habitats are concerning for this species, as they will likely negatively affect its population [100]. Similarly, the vulture *G. tenuirostris* is likely to lose 53–96% of its suitable habitation in future climate scenarios. The habitat quality has also deteriorated, with a 70.20% decrease in the number of patches in SSP245 and 77.25% reduction in SSP585 scenarios within the suitable habitats, resulting in a 98% decrease in patch size within its habitats. Apart from the drug diclofenac, vultures are also threatened by other factors such as habitat loss, food scarcity and electrocutions from power lines [101]. This trend of shrinking suitable habitats may push this critically endangered (CR) species to further decline. In future climate scenarios, it was observed that *H. leucoryphus* lost nearly 94–99% of its suitable habitats. The species showed a 77.05% decrease in the number of patches (NP) in SSP245 and 90.16% reduction in SSP585 climate scenarios, with a severe 99% reduction in patch size (Table 4). This species is primarily dependent on wetlands for prey, and it builds nests in tall trees near wetlands. The key threats to this species are habitat loss, degradation of wetlands, and over fishing [102]. Hence, this alarming trend of habitat loss is critical for this endangered (EN) species.

According to research and conservation priority index [35], *F. severus*, *G. tenuirostris* and *H. leucoryphus* are the three priority raptors for conservation, which all breed in northeastern India. However, the region has also faced about 60% forest cover loss within a span of the last twenty years (www.globalforestwatch.org/ accessed on 5 June 2025). In addition, studies have reported a declining trend in monsoon precipitation in Northeast India over the last few decades [103]. Such a trend might lead to the loss of wetlands, which are a primary source of prey for species like *H. leucoryphus.* Also, land use change is a multifaceted issue in Northeast India, influenced by many socio-economic and environmental factors. Rapid urbanization, shifting cultivations, monocultural plantations like tea and oil palm, etc., have led to a decline in the forest lands in the region [104,105]. Although vultures have been the most studied group of raptors in the region, *F. severus* and *H. leucoryphus* have received negligible research attention. Furthermore, there has been almost no research on the impact of climate change and habitat loss on these three raptors. As per the SoIB (2023) [44], there are insufficient data on the current annual trend and long-term trend in the population for *F. severus* in India. Also, for *G. tenuirostris*, there is inadequate data on its population trend. And the current trend in *H. leucoryphus* suggests a rapid decline in the population.

Across the Indian subcontinent, the dramatic decline in vultures has resulted in notable increases in mammalian scavenger populations, particularly rats (*Rattus* sp.) and feral dogs (*Canis lupus familiaris*) [106,107]. These species act as key reservoirs of pathogens responsible for diseases such as bubonic plague and rabies. Consequently, the public health costs associated with the loss of vultures’ regulating ecosystem services have been estimated to exceed USD 2 billion annually [107]. The decomposition of carcasses also enhances nutrient inputs into soils [108], with potential cascading effects on microbial communities, vegetation, and invertebrate assemblages. Moreover, both diurnal and nocturnal raptors, as avian predators, are considered pivotal in regulating rodent population dynamics [109,110]. Designating raptors as “flagship species” ensures that conserving their habitats benefits many, though not all, co-occurring species and ecosystems, thereby enhancing the cost-effectiveness of conservation investments and advancing global environmental objectives [111]. Shifting species distributions can generate novel assemblages that modify ecosystem processes, such as prey regulation and resource competition, with outcomes that remain difficult to anticipate due to the complexity of interacting variables [112]. Such changes in community structure are expected to disproportionately affect specialists [113,114] and species with narrow ranges, particularly those confined to polar or alpine environments where contractions are projected, for instance, the tundra biome, and may diminish by up to 34% [115]. Climate change is expected to have a severe impact on birds, and hence it is imperative to assess the effectiveness of conservation programs by comprehending the current and projected effect of the changing climate [116].

The areas of high suitability for *F. severus* in Assam are primarily in the BTR regions and Upper Assam areas. These regions need focused conservation actions such as enhancement of community-level forest protection programs at the forest–edge interface to reduce habitat alteration; the creation of buffer areas and green corridors connecting fragmented patches, especially between the BTR regions and adjacent foothill forests; and the use of pesticide is agricultural areas, which are used by raptors as foraging grounds, should be monitored and regulated. The key areas of distribution of *G. tenuirostris* mostly lie within the upper Assam and eastern Arunachal. Assam has a Vulture Breeding Centre, which plays a vital role in establishing a good reintroduced vulture population in the wild. The success of species reintroduction programs relies heavily on knowledge of their climatic and habitat requirements, which is particularly vital for the conservation of critically endangered vultures. The subsequent stage involves releasing captive-bred individuals into climatically stable, diclofenac-free areas or officially designated vulture safe zones. Therefore, identifying suitable climatic niches is fundamental to ensuring the effective reintroduction of vultures into the wild. Food source availability is one of the key drivers of vulture population. Supplementary feeding approaches such as “vulture restaurants” have been employed to sustain populations, signifying the importance of reliable food bases in vulture conservation. Research has shown that supplying uncontaminated food through vulture restaurants can help lower diclofenac-related mortality within vulture colonies [117]. Tracking data from tagged vultures revealed that the restaurant also influenced foraging behavior, leading to smaller home ranges, reduced flight time, and shorter daily travel distances once the predictable food source was available [117]. *H. leucoryphus* migrates to the Northeastern region in winter for breeding [118]. The conservation of forest areas near wetlands is important for their nesting. The water bodies in the Brahmaputra plains and the foothills of eastern Himalayas need to be conserved, as they act as prey resources for this species. To safeguard this migratory raptor, transboundary collaboration will play a key role. The different threats and conservation implications have been added in a tabular form (Table 5).

## 5. Limitations

This study is subject to several important limitations, primarily linked to the application of SDMs. These models generate predictive results that are highly dependent on the quality, completeness, and accuracy of the input data. Consequently, even small changes in input parameters may lead to slight variations in the results, while the overall distribution patterns and trends typically remain consistent. Additionally, the pseudo-absences were generated randomly across the study area which might influence the suitability estimates. The study used conventional k-fold cross-validations which might also inflate the model evaluation, when there is spatial clustering of occurrence data. Hence, future studies can apply block or spatial cross-validations approach is suggested to deal with spatially clustered occurrence data. While the raptors considered in this study have broad distributions across the Northeast region, confirmed occurrence records are largely concentrated within protected areas, where detections are more frequent, often facilitated by citizen science initiatives. Therefore, targeted large-scale surveys are recommended across the landscape to identify more areas of species presence. Furthermore, the study used current habitat data to project future environment suitability, given the high uncertainty surrounding future land cover dynamics. Consequently, the projections do not incorporate probable shifts in land use that may occur in the future. The distributional changes in species ranges observed in this study are therefore accredited only to climatic variables. Although this approach provides important insights into climate-driven range alterations, it may underrate or overlook the combined effects of land use change. Hence, future studies should try to integrate land cover projections alongside climatic variables to produce more comprehensive and ecologically realistic assessments. Furthermore, relying on a single GCM may not fully capture the variability among future climate projections. Therefore, future studies are recommended to incorporate multiple GCMs for each species to improve predictive accuracy and better identify potential areas of species presence. Thus, building on the foundation established by this study, such approaches can provide more detailed and robust insights for conservation planning.

## 6. Conclusions

All three species have a restricted distribution range within the eastern Himalayas and the northeast biogeographic region of India. Our findings highlight that climate change and topographical restraints could potentially diminish the suitable habitat ranges for the studied species. Hence, our study predicts the fate of these important bird species and their vulnerability to the inevitable climate change effect in the future. In Northeast India, priority needs to be given to protect the Eastern Himalaya foothill regions, which include important wetlands, lowland forests and contiguous habitats, and which provide refuge to different raptors. These species are already threatened by anthropogenic pressures and may be further vulnerable to environmental stress. Hence, the effects of changing climate on their limiting factors, such as nesting biology, habitat ecology, and food resources, etc., need long-term assessment for effective conservation actions. With more extreme weather conditions expected in the future, evaluation of species-specific traits such as food habits, nest type, breeding phenology, body size, etc., that are linked to species’ resistance to climate change, is highly important. Through this study, the need for revaluation of the status of these species and the inclusion of climate change and habitat fragmentation for conservation threat assessments is also emphasized.

## Figures and Tables

**Table 1:** Model fit metrics for the used models and for the ensemble model of habitat suitability evaluation of the three raptors. The five modeling algorithms are: Boosted Regression Tree (BRT), Generalized Linear Model (GLM), Multivariate Adaptive Regression Splines (MARS), Maximum Entropy (MaxEnt), and Random Forest (RF). AUC: Area under Curve, ΔAUC: Change in Area under Curve (Training–Cross Validation), PCC: Proportion Correctly Classified, TSS: True Skill Statistic.

Species	Model	Dataset	AUC	ΔAUC	PCC	TSS	Kappa	Specificity	Sensitivity
*Falco severus*	BRT	Train	0.921	0.053	85.500	0.852	0.860	0.711	0.709
		CV	0.868		80.000	0.845	0.747	0.592	0.593
	GLM	Train	0.951	0.122	88.100	0.883	0.879	0.761	0.760
		CV	0.829		76.500	0.776	0.750	0.526	0.525
	MARS	Train	0.910	0.066	83.000	0.828	0.832	0.660	0.658
		CV	0.844		79.600	0.819	0.769	0.588	0.588
	MaxEnt	Train	0.934	0.062	87.600	0.874	0.879	0.753	0.751
		CV	0.872		79.500	0.825	0.762	0.587	0.584
	RF	Train	0.908	0.017	85.100	0.852	0.850	0.702	0.700
		CV	0.891		82.500	0.860	0.786	0.647	0.648
*Gyps* *tenuirostris*	BRT	Train	0.985	0.068	92.700	0.924	0.935	0.859	0.838
		CV	0.917		86.100	0.871	0.845	0.715	0.693
	GLM	Train	0.926	0.058	85.200	0.848	0.860	0.708	0.677
		CV	0.868		82.500	0.836	0.808	0.644	0.619
	MARS	Train	0.955	0.053	87.900	0.879	0.879	0.758	0.733
		CV	0.902		84.600	0.849	0.845	0.694	0.664
	MaxEnt	Train	0.948	0.041	89.700	0.897	0.897	0.794	0.772
		CV	0.907		84.500	0.853	0.835	0.688	0.662
	RF	Train	0.934	0.003	85.200	0.853	0.850	0.703	0.675
		CV	0.937		88.200	0.907	0.835	0.741	0.734
*Haliaeetus* *leucoryphus*	BRT	Train	0.988	0.030	93.300	0.933	0.933	0.866	0.866
		CV	0.958		88.600	0.874	0.900	0.774	0.772
	GLM	Train	0.955	0.022	90.900	0.911	0.908	0.819	0.818
		CV	0.933		87.900	0.890	0.867	0.756	0.759
	MARS	Train	0.970	0.038	92.500	0.926	0.924	0.850	0.850
		CV	0.932		90.600	0.911	0.900	0.811	0.811
	MaxEnt	Train	0.978	0.023	92.500	0.926	0.924	0.850	0.849
		CV	0.955		90.500	0.904	0.908	0.811	0.810
	RF	Train	0.957	0.001	89.400	0.787	0.787	0.891	0.896
		CV	0.958		89.400	0.789	0.787	0.900	0.889

**Table 2:** The mean contribution in percentage of different covariate used in the ensemble model for raptors.

Species	Predictors	BRT	GLM	MARS	MaxEnt	RF	μ (Mean)	μ (Mean) %
*Falco severus*	aspect	0.000	0.007	0.005	0.017	0.000	0.006	1.030
	bio15	0.000	0.285	0.060	0.098	0.003	0.089	15.390
	bio18	0.000	0.222	0.000	0.052	0.001	0.055	9.490
	bio19	0.289	0.315	0.139	0.077	0.023	0.169	29.020
	bio2	0.000	0.128	0.074	0.096	0.010	0.062	10.630
	bio4	0.000	0.087	0.059	0.103	0.001	0.050	8.580
	elevation	0.139	0.195	0.170	0.165	0.019	0.137	23.660
	slope	0.000	0.000	0.000	0.061	0.002	0.013	2.210
*Gyps tenuirostris*	aspect	0.000	0.004	0.008	0.006	0.001	0.004	1.110
	bio15	0.019	0.000	0.058	0.017	0.007	0.020	5.960
	bio18	0.017	0.052	0.000	0.017	0.004	0.018	5.250
	bio19	0.000	0.000	0.000	0.000	0.000	0.000	0.110
	bio2	0.020	0.036	0.075	0.028	0.006	0.033	9.620
	bio4	0.081	0.184	0.048	0.077	0.007	0.079	23.180
	elevation	0.082	0.254	0.052	0.187	0.061	0.127	37.140
	slope	0.017	0.057	0.076	0.016	0.005	0.034	9.940
	bio14	0.000	0.017	0.055	0.059	0.000	0.026	7.700
*Haliaeetus leucoryphus*	aspect	0.000	0.000	0.000	0.001	0.000	0.000	0.130
	bio15	0.000	0.081	0.022	0.004	0.001	0.022	7.430
	bio18	0.000	0.000	0.000	0.000	0.000	0.000	0.120
	bio19	0.000	0.000	0.000	0.000	0.000	0.000	0.120
	bio2	0.000	0.000	0.007	0.020	0.001	0.006	1.960
	bio4	0.028	0.093	0.034	0.019	0.000	0.035	11.940
	elevation	0.286	0.241	0.056	0.149	0.056	0.158	54.020
	slope	0.000	0.000	0.060	0.035	0.002	0.019	6.670
	bio14	0.046	0.064	0.044	0.085	0.000	0.048	16.410
	bio16	0.000	0.000	0.016	0.000	0.001	0.003	1.190

**Table 3:** The suitable habitat extent (km^2^) of the three raptors in present and future climate scenarios. The “-” sign denotes loss in habitat area.

Species	Present	SSP245 (2041–2060)	Habitat Loss from Present in SSP245 (2041–2060) (%)	SSP245 (2061–2080)	Habitat Loss from Present in SSP245 (2061–2080) (%)	SSP585 (2041–2060)	Habitat Loss from Present in SSP585 (2041–2060) (%)	SSP585 (2061–2080)	Habitat Loss from Present in SSP585 (2061–2080) (%)
*Falco severus*	26,801	17,109	−36.16	9231	−65.55	12,184	−54.53	8389	−68.69
*Gyps* *tenuirostris*	29,297	13,731	−53.13	989	−96.62	6160	−78.97	919	−96.86
*Haliaeetus leucoryphus*	17,808	1021	−94.26	444	−97.50	419	−97.64	119	−99.33

**Table 4:** Habitat quality assessment and shape geometry of the raptors in present and future climate conditions. NP: Number of patches; LPI: Largest patch index; TE: Total Edge; LSI: Landscape shape index; AI: Aggregate Index.

Species	Scenario	NP	LPI	TE	LSI	AI
*Falco severus*	Present	200	5.091	80.368	15.314	91.187
	SSP245 (2041–2060)	303	3.198	75.392	17.985	86.895
	SSP245 (2061–2080)	134	1.816	43.696	14.150	86.108
	SSP585 (2041–2060)	212	2.517	50.096	14.167	87.949
	SSP585 (2061–2080)	118	1.051	39.184	13.310	86.351
*Gyps tenuirostris*	Present	255	8.371	84.640	15.423	91.507
	SSP245 (2041–2060)	203	3.637	53.040	14.106	88.688
	SSP245 (2061–2080)	76	0.132	8.080	8.016	76.919
	SSP585 (2041–2060)	185	0.668	38.736	15.420	81.386
	SSP585 (2061–2080)	58	0.089	7.712	7.902	76.308
*Haliaeetus* *leucoryphus*	Present	61	4.998	41.024	9.603	93.502
	SSP245 (2041–2060)	46	0.171	8.512	8.313	76.340
	SSP245 (2061–2080)	14	0.003	0.944	4.214	39.189
	SSP585 (2041–2060)	8	0.002	0.432	3	37.931
	SSP585 (2061–2080)	6	0.002	0.401	2.330	32.681

**Table 5:** Threat and conservation implications of the raptors in the present study.

Species	Threats (IUCN)	Necessary Responses	Scale of Action
*Falco severus*	Climate change, habitat loss and fragmentation. The percentage of suitable area loss from present area is 36.16% during 2041–2060 (SSP245), and loss during 2061–2080 (SSP245) is 65.55%. Similarly, the percent suitable area loss during 2041–2060 (SSP585) is 54.53%, and there is 68.69% loss during 2061–2080 (SSP585).	Long-term population trend monitoring by citizen science program. Impact assessment. Strict law enforcement. Awareness and education. Climate change adaptive management.	Regional
*Gyps tenuirostris*	Climate change, habitat loss and fragmentation. The percentage of suitable area loss from present area is 53.13% during 2041–2060 (SSP245), and loss during 2061–2080 (SSP245) is 96.62%. Similarly, the percent suitable area loss during 2041–2060 (SSP585) is 78.97%, and there is 96.86% loss during 2061–2080 (SSP585).	Updated vulture action plan. Updated areas of vulture safe zones. Awareness and advocacy. Long-term population trend monitoring by citizen science program. Impact assessment. Climate change adaptive management.	Regional
*Haliaeetus leucoryphus*	Climate change, Habitat loss and fragmentation. The percentage of suitable area loss from present area is 94.26% during 2041–2060 (SSP245), and loss during 2061–2080 (SSP245) is 97.5%. Similarly, the percent suitable area loss during 2041–2060 (SSP585) is 97.64% and there is 99.33% loss during 2061–2080 (SSP585).	Identification of suitable sites for protection. Recommend sustainable wetland management practices. Regulate pollution of wetlands. Nesting trees plantation around waterbodies. Conduct education and awareness programs in rural areas having wetlands. Long-term population trend monitoring by citizen science program. Impact assessment.	Regional and International

## Data Availability

The original contributions presented in this study are included in the article/Supplementary Material. Further inquiries can be directed to the corresponding author(s).

## Supplementary Materials

The following supporting information can be downloaded at: https://www.mdpi.com/article/10.3390/biology14101376/s1, Figure S1. Final set of variables retained for ensemble model approach after excluding highly correlated covariates. The figure illustrates the pairwise correlations (|r| < 0.8) among variables selected for *Falco severus*. The Pearson’s correlation coefficient is used as the primary measure. If either the Spearman or Kendall coefficient exceeds the Pearson value for a given pair, it is indicated with an “s” (Spearman) or “k” (Kendall) in the bottom-right corner of the corresponding cell. Figure S2. Final set of variables retained for ensemble model approach after excluding highly correlated covariates. The figure illustrates the pairwise correlations (|r| < 0.8) among variables selected for *Gyps tenuirostris*. The Pearson’s correlation coefficient is used as the primary measure. If either the Spearman or Kendall coefficient exceeds the Pearson value for a given pair, it is indicated with an “s” (Spearman) or “k” (Kendall) in the bottom-right corner of the corresponding cell. Figure S3. Final set of variables retained for ensemble model approach after excluding highly correlated covariates. The figure illustrates the pairwise correlations (|r| < 0.8) among variables selected for *Haliaeetus leucoryphus*. The Pearson’s correlation coefficient is used as the primary measure. If either the Spearman or Kendall coefficient exceeds the Pearson value for a given pair, it is indicated with an “s” (Spearman) or “k” (Kendall) in the bottom-right corner of the corresponding cell. Figure S4. The ROC plots and variable importance analyses for five SDM algorithms applied to *Falco severus*: (A) BRT, (B) GLM, (C) MARS, (D) MaxEnt, and (E) RF. The left panels present ROC curves indicating model performance for both training and cross-validation datasets, along with the corresponding AUC values. The right panels display the relative importance of environmental predictors as determined by each model. All graphs were generated using the SAHM (Software for Assisted Habitat Modeling) package integrated within the VisTrails platform and manually refined in Adobe Photoshop CS 8.0. Figure S5. The ROC plots and variable importance analyses for five SDM algorithms applied to *Gyps tenuirostris*: (A) BRT, (B) GLM, (C) MARS, (D) MaxEnt, and (E) RF. The left panels present ROC curves indicating model performance for both training and cross-validation datasets, along with the corresponding AUC values. The right panels display the relative importance of environmental predictors as determined by each model. All graphs were generated using the SAHM (Software for Assisted Habitat Modeling) package integrated within the VisTrails platform and manually refined in Adobe Photoshop CS 8.0. Figure S6. The ROC plots and variable importance analyses for five SDM algorithms applied to *Haliaeetus leucoryphus*: (A) BRT, (B) GLM, (C) MARS, (D) MaxEnt, and (E) RF. The left panels present ROC curves indicating model performance for both training and cross-validation datasets, along with the corresponding AUC values. The right panels display the relative importance of environmental predictors as determined by each model. All graphs were generated using the SAHM (Software for Assisted Habitat Modeling) package integrated within the VisTrails platform and manually refined in Adobe Photoshop CS 8.0. Table S1. This table lists all the initial variables considered in the study, along with their categories and data sources, prior to performing correlation analysis for variable selection. Table S2. The table shows the suitable area (in km^2^) within the study area for the three raptor species under present and future climatic scenarios.
