# Supplementary material for: Rulers of the Open Sky at Risk: Climate-Driven Habitat Shifts of Three Conservation-Priority Raptors in the Eastern Himalayas
Source: Biology (Basel). 2025 Oct 8;14(10):1376. doi: 10.3390/biology14101376 (PMC12562076; doi:10.3390/biology14101376)
Supplement: Supplementary file 1 [file biology-14-01376-s001.zip › biology-3839566-supplementary.pdf]

Supplementary Materials

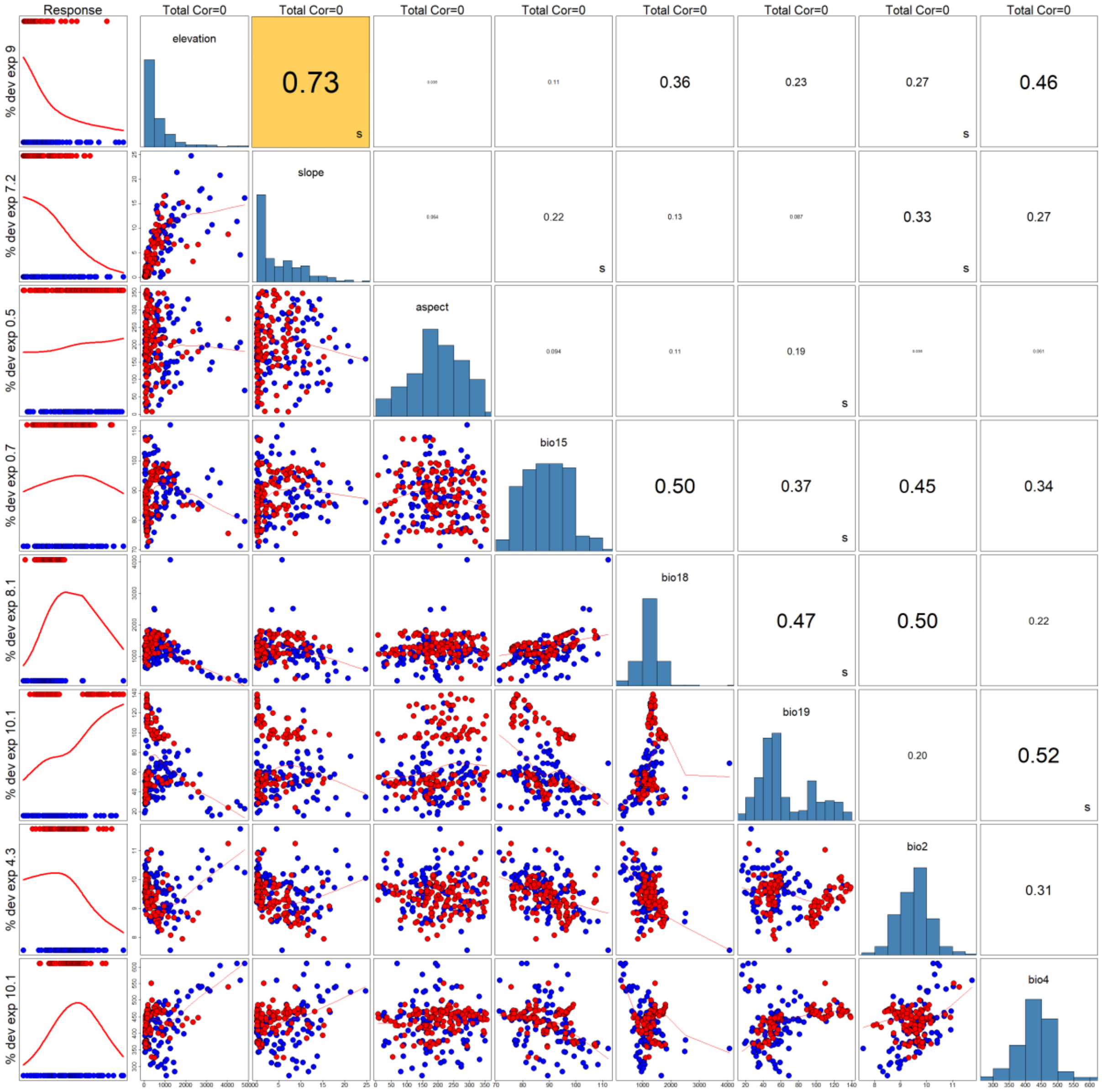

Figure S1. Final set of variables retained for ensemble model approach after excluding highly correlated covariates. The figure illustrates the pairwise correlations (|r| < 0.8) among variables selected for *Falco severus*. The Pearson's correlation coefficient is used as the primary measure. If either the Spearman or Kendall coefficient exceeds the Pearson value for a given pair, it is indicated with an "s" (Spearman) or "k" (Kendall) in the bottom-right corner of the corresponding cell.

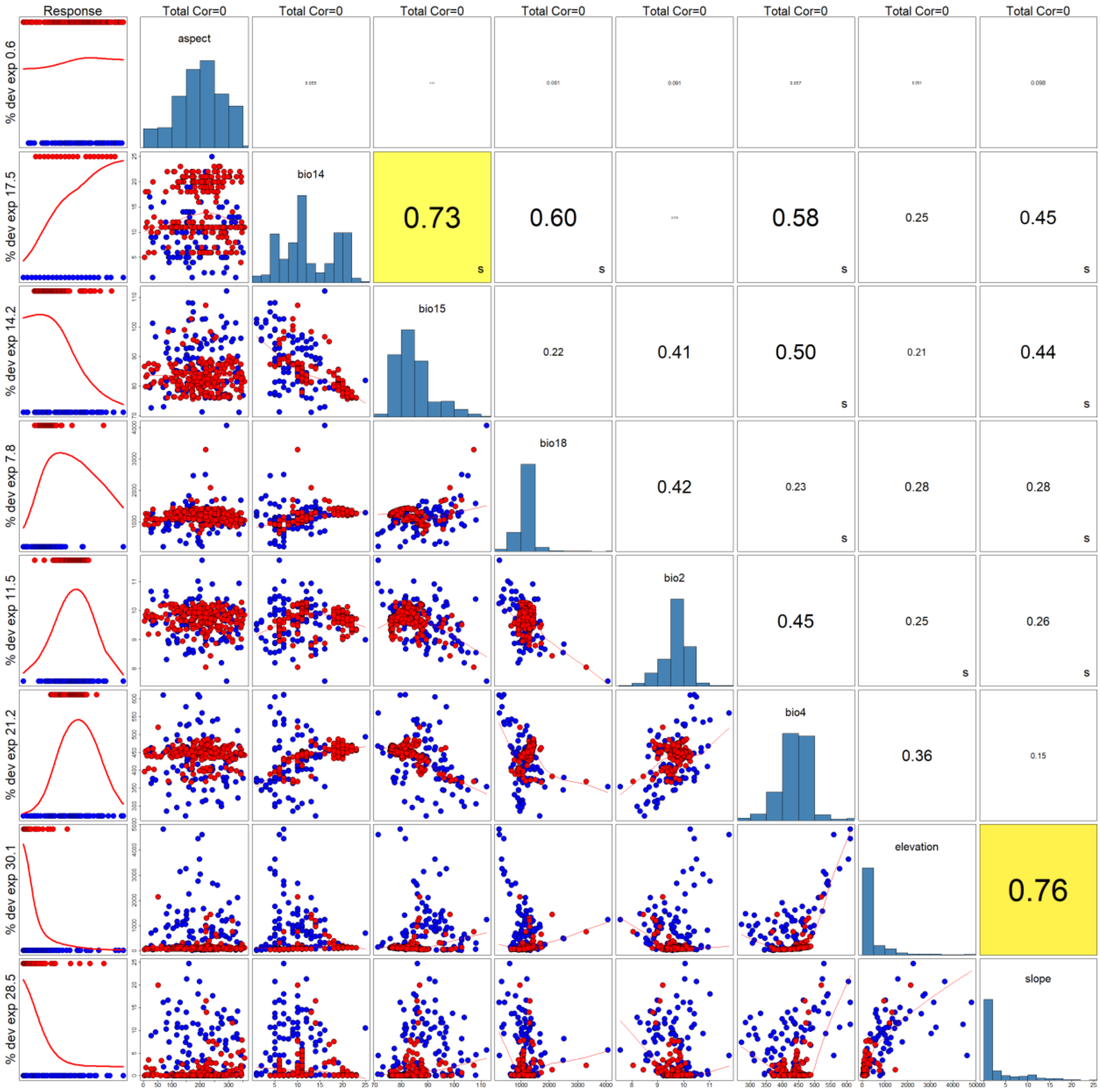

Figure S2. Final set of variables retained for ensemble model approach after excluding highly correlated covariates. The figure illustrates the pairwise correlations ($|r| < 0.8$) among variables selected for *Gyps tenuirostris*. The Pearson's correlation coefficient is used as the primary measure. If either the Spearman or Kendall coefficient exceeds the Pearson value for a given pair, it is indicated with an "s" (Spearman) or "k" (Kendall) in the bottom-right corner of the corresponding cell.

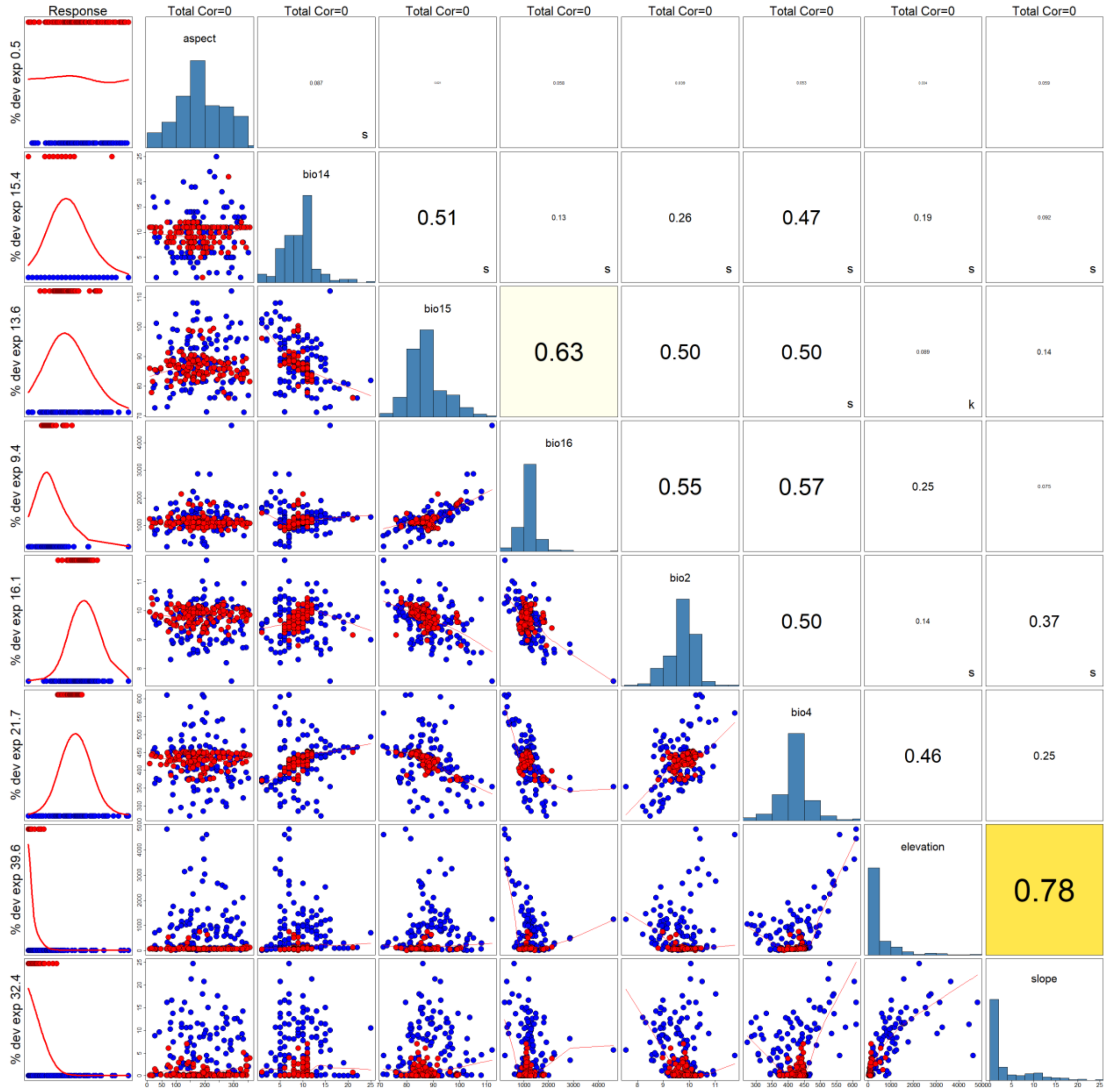

Figure S3. Final set of variables retained for ensemble model approach after excluding highly correlated covariates. The figure illustrates the pairwise correlations (|r| < 0.8) among variables selected for *Haliaeetus leucoryphus*. The Pearson's correlation coefficient is used as the primary measure. If either the Spearman or Kendall coefficient exceeds the Pearson value for a given pair, it is indicated with an "s" (Spearman) or "k" (Kendall) in the bottom-right corner of the corresponding cell.

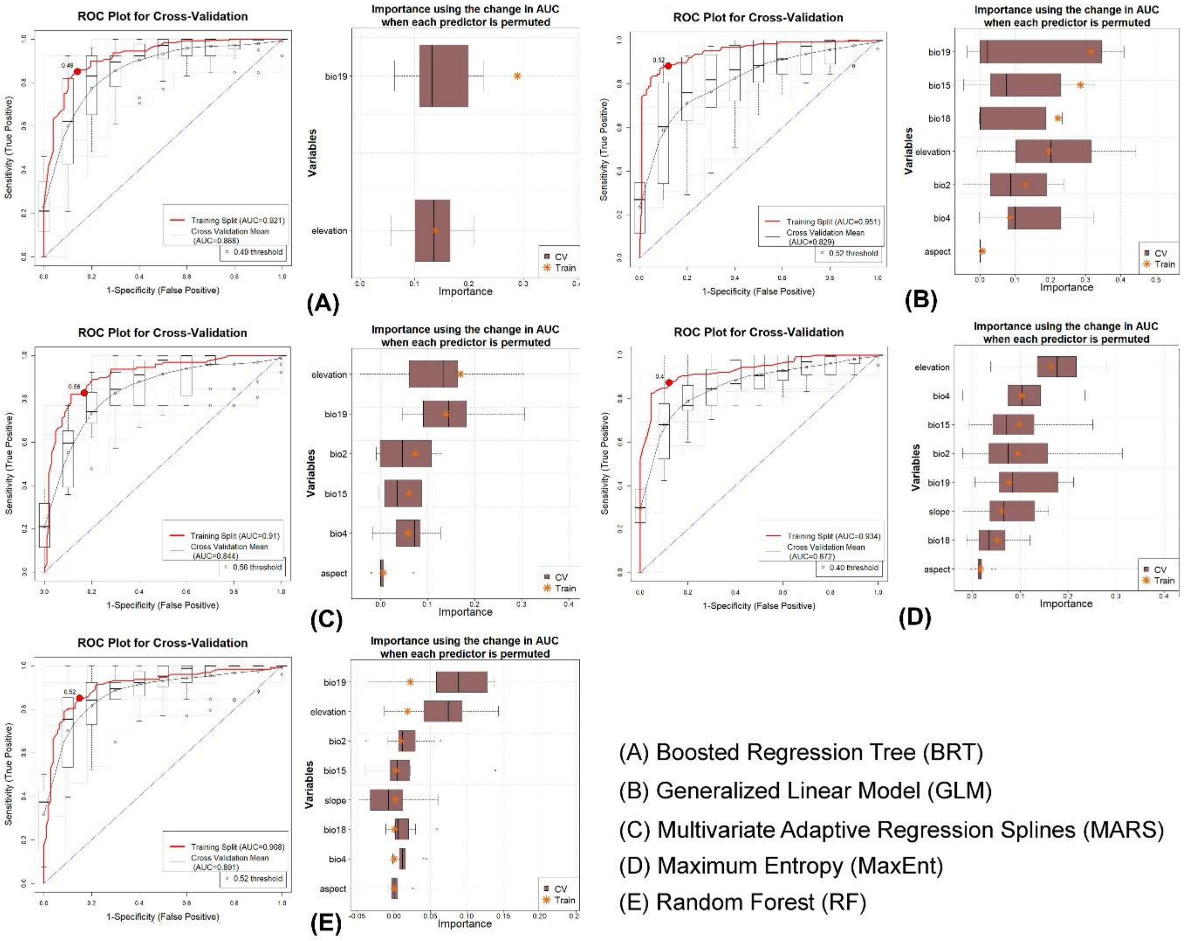

Figure S4. The ROC plots and variable importance analyses for five SDM algorithms applied to *Falco severus*: (A) BRT, (B) GLM, (C) MARS, (D) MaxEnt, and (E) RF. The left panels present ROC curves indicating model performance for both training and cross-validation datasets, along with the corresponding AUC values. The right panels display the relative importance of environmental predictors as determined by each model. All graphs were generated using the SAHM (Software for Assisted Habitat Modeling) package integrated within the VisTrails platform and manually refined in Adobe Photoshop CS 8.0.

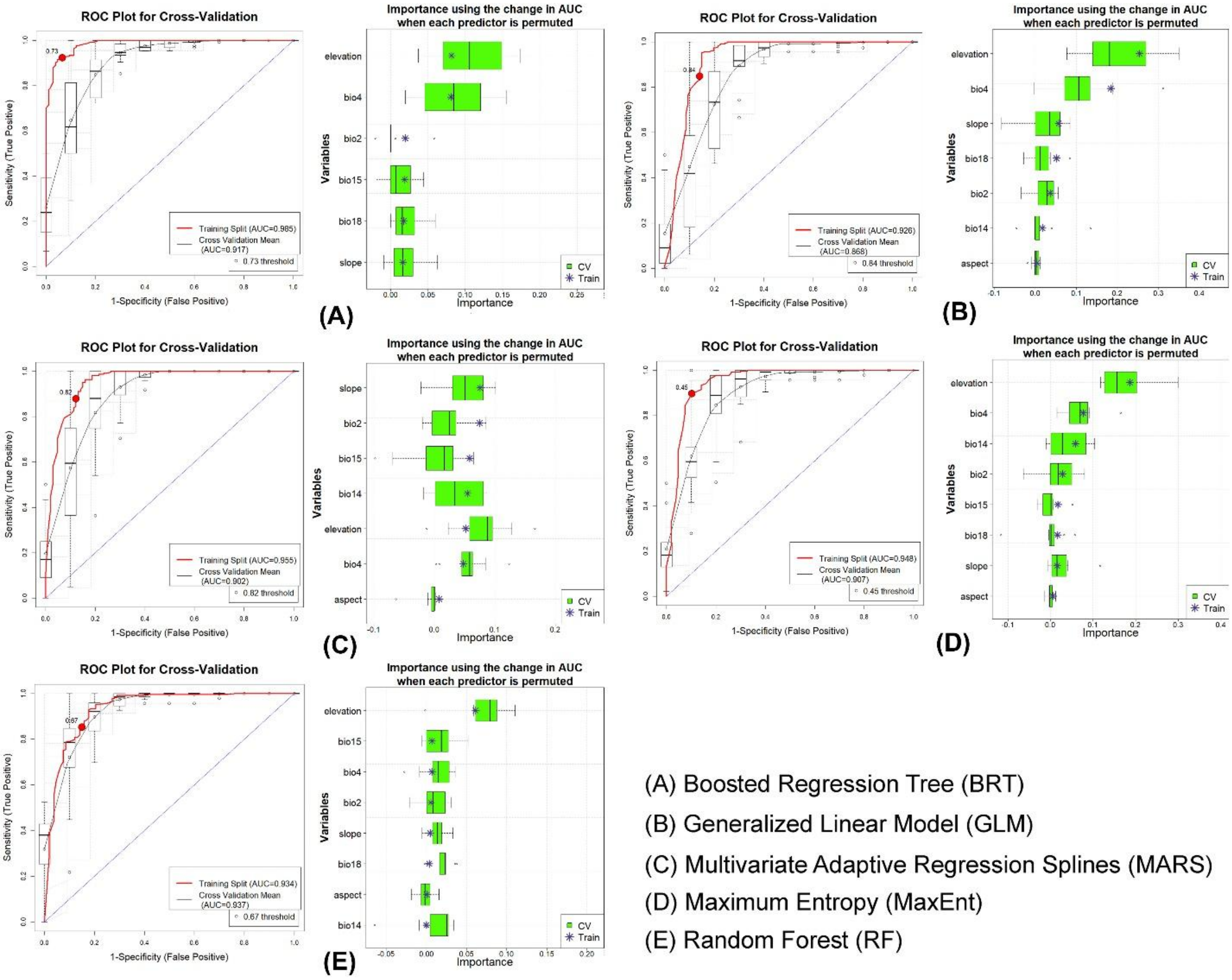

Figure S5. The ROC plots and variable importance analyses for five SDM algorithms applied to *Gyps tenuirostris*: (A) BRT, (B) GLM, (C) MARS, (D) MaxEnt, and (E) RF. The left panels present ROC curves indicating model performance for both training and cross-validation datasets, along with the corresponding AUC values. The right panels display the relative importance of environmental predictors as determined by each model. All graphs were generated using the SAHM (Software for Assisted Habitat Modeling) package integrated within the VisTrails platform and manually refined in Adobe Photoshop CS 8.0.

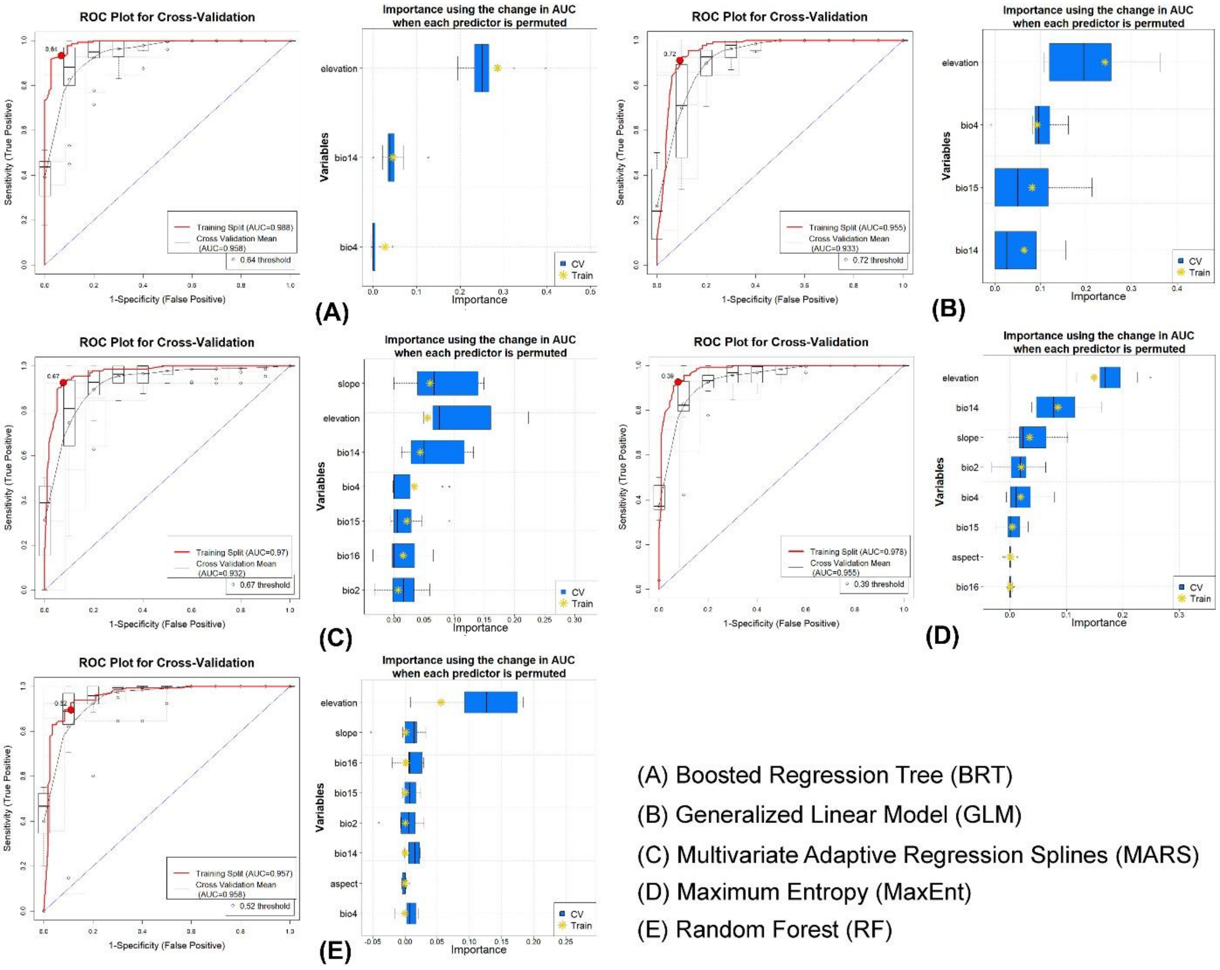

Figure S6. The ROC plots and variable importance analyses for five SDM algorithms applied to *Haliaeetus leucoryphus*: (A) BRT, (B) GLM, (C) MARS, (D) MaxEnt, and (E) RF. The left panels present ROC curves indicating model performance for both training and cross-validation datasets, along with the corresponding AUC values. The right panels display the relative importance of environmental predictors as determined by each model. All graphs were generated using the SAHM (Software for Assisted Habitat Modeling) package integrated within the VisTrails platform and manually refined in Adobe Photoshop CS 8.0.

Table S1. This table lists all the initial variables considered in the study, along with their categories and data sources, prior to performing correlation analysis for variable selection.

Category	Abbreviation	Variables	Source
Bioclimatic	bio_1	Annual Mean Temperature	https://www.worldclim.org/
	bio_2	Mean Diurnal Range (Mean of monthly (max temp - min temp))	https://www.worldclim.org/
	bio_3	Isothermality (Bio2/Bio7)	https://www.worldclim.org/
	bio_4	Temperature Seasonality (Standard deviation of temperature)	https://www.worldclim.org/
	bio_5	Max Temperature of Warmest Month	https://www.worldclim.org/
	bio_6	Min Temperature of Coldest Month	https://www.worldclim.org/
	bio_7	Temperature Annual Range (Bio5 - Bio6)	https://www.worldclim.org/
	bio_8	Mean Temperature of Wettest Quarter	https://www.worldclim.org/
	bio_9	Mean Temperature of Driest Quarter	https://www.worldclim.org/
	bio_10	Mean Temperature of Warmest Quarter	https://www.worldclim.org/
	bio_11	Mean Temperature of Coldest Quarter	https://www.worldclim.org/
	bio_12	Annual Precipitation	https://www.worldclim.org/
	bio_13	Precipitation of Wettest Month	https://www.worldclim.org/
	bio_14	Precipitation of Driest Month	https://www.worldclim.org/
	bio_15	Precipitation Seasonality (Coefficient of Variation)	https://www.worldclim.org/
	bio_16	Precipitation of Wettest Quarter	https://www.worldclim.org/
	bio_17	Precipitation of Driest Quarter	https://www.worldclim.org/
	bio_18	Precipitation of Warmest Quarter	https://www.worldclim.org/
	bio_19	Precipitation of Coldest Quarter	https://www.worldclim.org/
Topographic	elevation	Elevation	http://srtm.csi.cgiar.org/srtmdata/
	slope	Slope	http://srtm.csi.cgiar.org/srtmdata/
	aspect	Aspect	http://srtm.csi.cgiar.org/srtmdata/

Table S2. The table shows the suitable area (in km^2) within the study area for the three raptor species under present and future climatic scenarios.

**Scenario**	***Falco severus***	***Gyps tenuirostris***	***Haliaeetus leucoryphus***
Present	26801	29297	17808
SSP245 (2041-2060)	17109	13731	1021
SSP245 (2061-2080)	9231	989	444
SSP585 (2041-2060)	12184	6160	419
SSP585 (2061-2080)	8389	919	119
